# Activating mutation of *PDGFRB* gene in a rare cardiac undifferentiated intimal sarcoma of the left atrium: a case report

**DOI:** 10.18632/oncotarget.20700

**Published:** 2017-09-07

**Authors:** Xiaoling Fu, Weixin Niu, Ji Li, Amber J. Kiliti, Hikmat A. Al-Ahmadie, Gopa Iyer, Sizhi Paul Gao, Qi Li

**Affiliations:** ^1^ Department of Medical Oncology, Shuguang Hospital, Shanghai University of Traditional Chinese Medicine, Shanghai, P.R. China; ^2^ Department of Surgery, Zhongshan Hospital of Fudan University, Shanghai, P.R. China; ^3^ Department of Pancreatic Surgery, Huashan Hospital, Fudan University and Shanghai Medical School, Shanghai, P.R. China; ^4^ Human Oncology and Pathogenesis Program, Memorial Sloan Kettering Cancer Center, New York, New York, USA; ^5^ Department of Pathology, Memorial Sloan Kettering Cancer Center, New York, New York, USA; ^6^ Department of Medicine, Memorial Sloan Kettering Cancer Center, New York, New York, USA; ^7^ Weill Cornell Medical College, Cornell University, New York, New York, USA

**Keywords:** cardiac sarcoma, intimal sarcoma, memorial sloan kettering-integrated mutation profiling of actionable cancer targets, PDGFRA, PDGFRB

## Abstract

Cardiac sarcoma is a rare malignant tumor with undefined genetic mutations and no targeted therapy. Here in one rare case of undifferentiated cardiac intimal sarcoma (IS), a next-generation sequencing based assay, MSK-IMPACT (Memorial Sloan Kettering - Integrated Mutation Profiling of Actionable Cancer Targets), identified a somatic, activating mutation in *PDGFRB*, along with amplification of *PDGFRA*. This E472D mutation of PDGFRB was discovered for the first time in IS. These findings suggest that concurrent aberrant PDGFRA and PDGFRB signaling may be a diagnostic biomarker and molecular therapeutic target of IS of the heart.

## INTRODUCTION

Primary malignant cardiac tumors are very rare, with an incidence of 0.001% ∼ 0.003%, with sarcomas comprising 75%. [1]. Cardiac sarcomas (CS) are often hard to detect until hemodynamic is compromised. The prognosis of CS is poor due to resistance to chemotherapy and radiation therapy, with a 5 to 15 month median survival. Surgical treatment is the only effective short-term management, but post-operative tumor recurrence and metastasis are common. Without a clearly characterized molecular mechanism for oncogenesis, targeted therapy for CS is unavailable. Studies of CS are scarce due to the low incidence, poor prognosis and short patient survival.

A recent study with hitherto the largest cohort of 100 primary CS cases has found that intimal sarcomas (IS) are the most frequent cardiac sarcoma histotype (∼42%) [2]. IS originates from subendothelial cells in the intima of large blood vessels of the central circulation system, often involving left atrium and mitral valve [2, 3]. IS usually appears to be a poorly differentiated sarcoma composed of atypical spindle and/or pleomorphic cells, with high degree of mitotic activity and possible myxoid areas [2, 3]. IS are heterogeneous and can differentiate into angio-, rhabdomyo-, or osteosarcomatous histotypes, making it difficult to be accurately diagnosed, and are therefore further divided into differentiated and undifferentiated subtypes. Previous molecular analysis of the few available IS cases revealed frequent concurrent amplification/activation of PDGFRA, EGFR and MDM2 [2–5].

Here, we describe a rare case of IS in a female patient's left atrium. Genomic screening of 410 key cancer genes using next-generation sequencing based MSK^TM^-IMPACT revealed somatic alterations in 3 important genes: *PDGFRB*, *KMT2D* and *MPL*, along with amplification or deletion of *PDGFRA*, *MDM*2, *KIT*, *CDKN2Ap16INK4A*, *CDKN2Ap14ARF* and *CDKN2B*. The E472D missense mutation of PDGFRB was identified for the first time in any tumor and was accompanied by elevated levels of activated/phosphorylated PDGFRB. This discovery provides a crucial piece of evidence suggesting that dual activation of PDGFR(A/B) signaling may be a biomarker and therapeutic target for IS.

## CASE PRESENTATION AND MOLECULAR PROFILING

**Figure 1:**
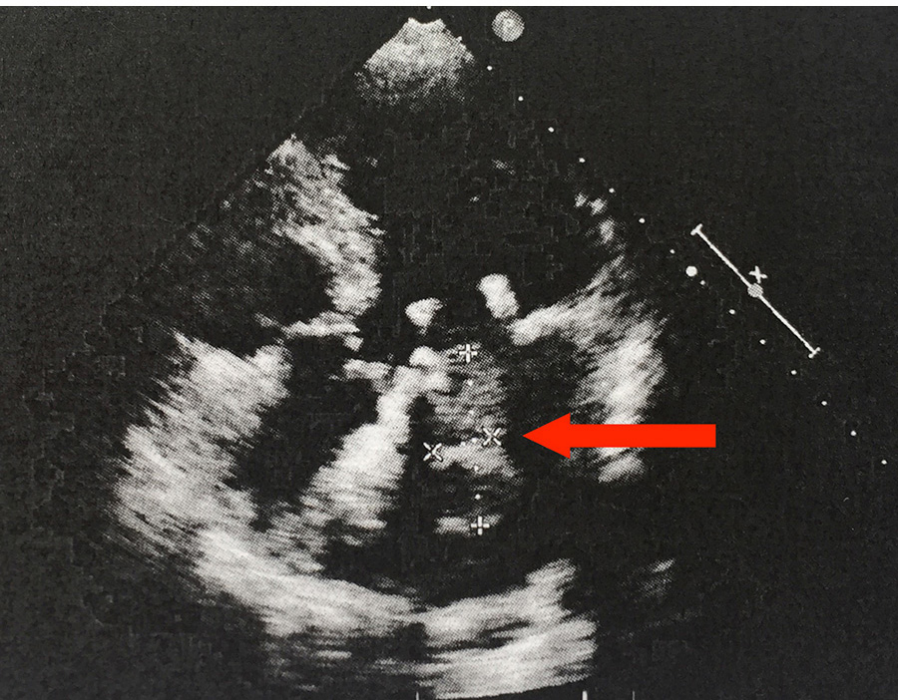
Echocardiogram, four-chamber view The 70-year-old female patient developed a space-occupying lesion in the left atrium. The echocardiogram shows a 4.05×1.39 cm echogenic mass attached to the mitral valve. The arrow indicates the mass.

A 70-year-old female presented with history of 2-month persistent cough and 1-month nocturnal orthopnea. Physical examination and echocardiography (Figure 1) indicated a space-occupying lesion, size 4.05×1.39 cm, in the left atrium. The patient went through procedures of complete surgical resection of the tumor, mitral valve replacement, tricuspid valve plasty and catheter radiofrequency ablation. The patient made an uneventful recovery with all pre-operative symptoms resolved. No post-surgical chemotherapy was given. The sarcoma recurred 5 months after the surgery. The patient has been on palliative treatment since and is still alive at the last follow-up.

**Figure 2:**
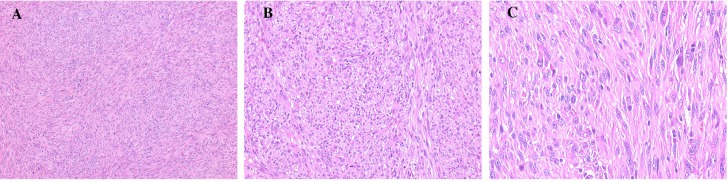
Histology of cardiac undifferentiated IS of this patient composed of spindle and pleomorphic cells (**A**) (x100), (**B**) (x200) and (**C**) (x400).

Pathologic examination confirmed the diagnosis of undifferentiated intimal sarcoma (UIS), with fibrous hyperplasia and myxomatosis of left mitral valve. The tumor, Grade 3 by Soft Tissue Tumor Differentiation Grading System, was composed of abundant atypical spindle and pleomorphic cells, with higher mitotic index (38/10 HPF), consistent with those seen in UIS (Figure 2). On immunohistochemical (IHC) examination, the sarcoma tumor cells were stained positive for CD34, CD31, vimentin, CD68, Calponin, F8 and Ki67 (80%), while negative for Bcl-2, Desmin, EMA, SMA, CK and ALK-1.

**Figure 3:**
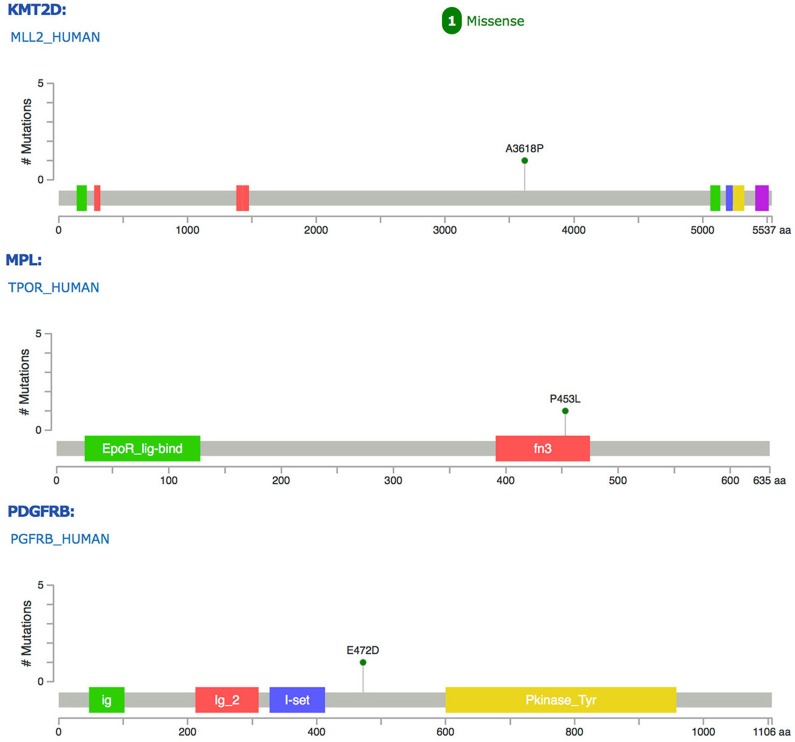
Mutations of PDGFRB, KMT2D and MPL genes identified in this case of cardiac IS Colored blocks represent functional domains of these gene products.

The tumor sample was subjected to MSK-IMPACT gene profiling. Surprisingly, somatic mutations involving only 4 genes, PDGFRB, KMT2D, MPL and ZFHX3, were identified, namely, E472D for PDGFRB, A3618P for KMT2D, P453L for MPL, and Q3202-Q3204dup/G3511-G3512del for ZFHX3 (Figure 3). By comparing against gnomAD.org human SNPs database, we excluded the ZFHX3 mutations as normal variants. In addition, we performed copy number analysis and found significantly altered genomic regions. The amplified regions were 12q15, harboring *MDM2*; 4q12, harboring *PDGFRA*, *KIT* and *KDR*; 19p13, harboring *DNMT1*, *KEAP1* and *MEF2B*; the copy number loss region was 9p21, harboring *CDKN2Ap16INK4A*, *CDKN2Ap14ARF* and *CDKN2B* (Supplementary Figure 1).

**Figure 4:**
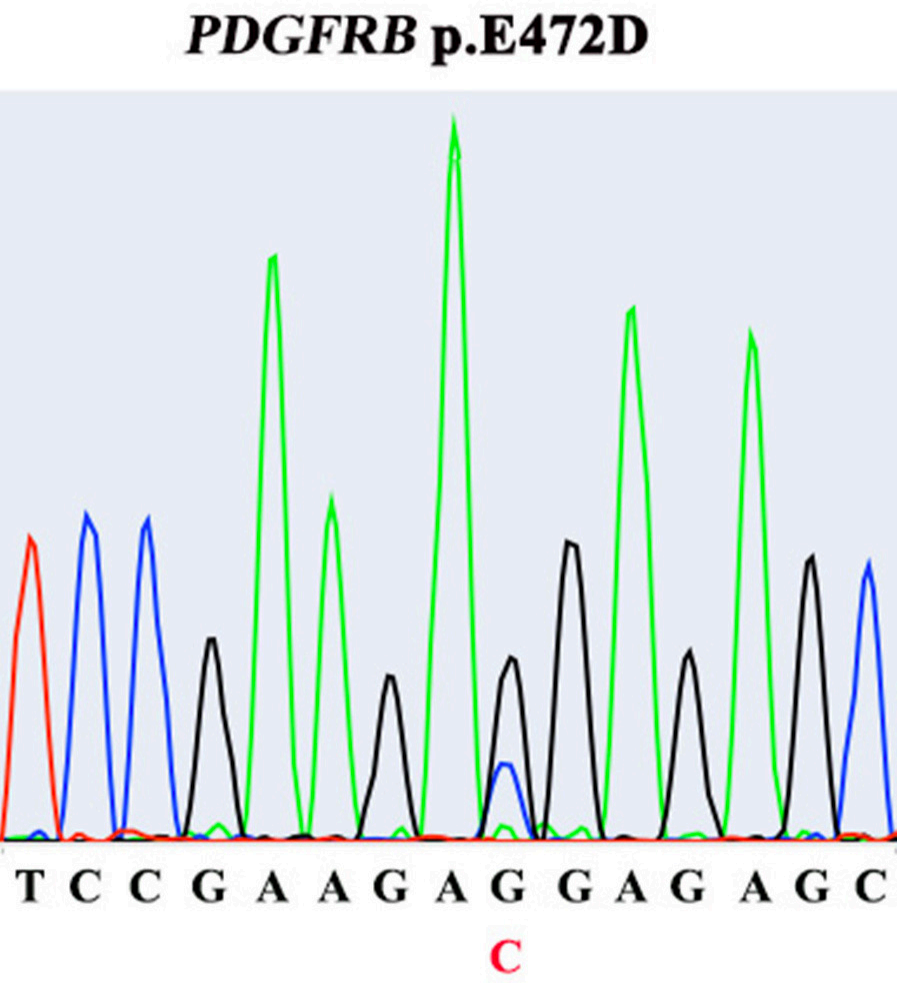
Presence of *PDGFRB* E472D mutation in tumor tissue, identified by Sanger sequencing

**Figure 5:**
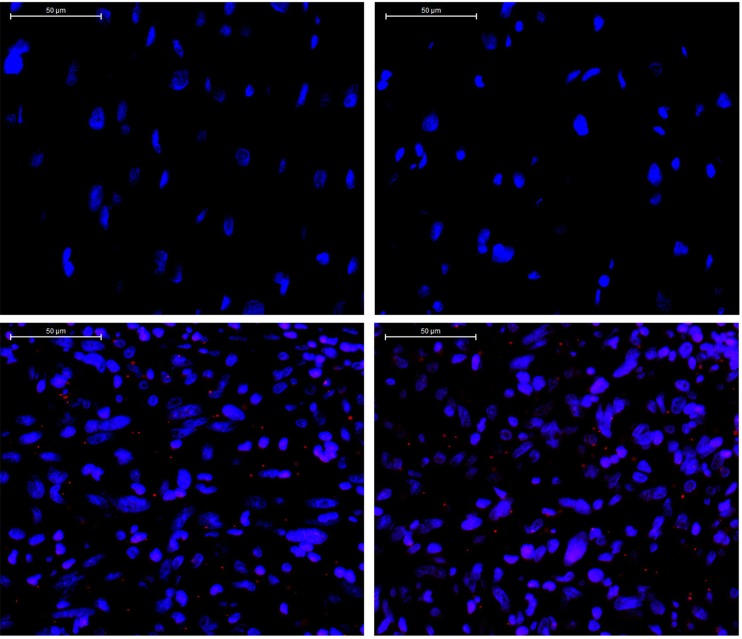
Detection of phosphorylated *PDGFRB* (*red dots*) by Duolink staining in tumor tissue Upper panel: two representative images of a stained control normal human heart tissue section. Lower panel: two representative images of a stained IS tissue section. (x400). DAPI: *blue*.

Aberrant PDGFR (A/B) signaling due to gene amplification has been suggested as a potential molecular signature of IS. One potentially activating point mutation of PDGFRB has recently been found [2–5]. Therefore, we decided to further explore the functionality of this PDGFRB mutation we identified in IS. The E472D mutation was validated using Sanger sequencing (Figure 4). Next, to examine the activation/phosphorylation status of PDGFRB (pPDGFRB) in IS tissue, we used *in situ* proximity ligase assay (PLA or Duolink), employing one primary rabbit monoclonal antibody to PDGFRB and the other mouse monoclonal antibody to phosphotyrosines on the same protein. Each antibody has established high specificity for their protein target [6]. This assay was chosen because: 1) Duolink has a high specificity for detection of pPDGFRB due to a dual recognition format, as shown previously [6]; 2) IHC and immunofluorescence staining using antibodies against pPDGFRB was low in sensitivity and specificity (data not shown) [6]; 3) we attained Duolink technology expertise as previously demonstrated [7]. Using this technique, we discovered pPDGFRB was present in the IS tumor section, but not in the normal control heart tissue (Figure 5) (Supplementary Figure 2).

## MATERIALS AND METHODS

### Clinical sample

Surgically resected tumor tissue was fixed in formalin, embedded in paraffin, sectioned, stained with hematoxylin and eosin (H&E) and reviewed by three pathologists independently. The diagnosis of intimal sarcoma (IS) was in accordance with the WHO and the recently proposed IS classification [8, 9]. Written informed consent was obtained from the patient The study is approved by Shuguang Hospital Affiliated to Shanghai University of Traditional Chinese Medicine Institutional Appraisal Committee.

### DNA extraction and exon capture next-generation sequencing

Tumors were profiled for genomic alterations in 410 key cancer-associated genes using our custom, deep sequencing MSK-IMPACT assay. Custom DNA probes were designed for targeted sequencing of all exons and selected introns of 410 oncogenes, tumor suppressor genes, and members of pathways deemed actionable by targeted therapies. Genomic DNA from the tumor, extracted on a Hamilton Chemagic workstation using formalin-fixed paraffin-embedded tissue DNA kits (Perkin Elmer), was subjected to sequence library preparation and exon capture (NimbleGen). Up to 30 barcoded sequence libraries were pooled at equimolar concentrations and input into a single exon capture reaction, as previously described [10]. Pooled libraries containing captured DNA fragments were subsequently sequenced on the Illumina HiSeq 2500 system as 2 × 100 bp paired-end reads. Sequence data were demultiplexed using BCL2FASTQv1.8.3 (Illumina), and vesitigial adapter sequences were removed from the 3′ end of sequence reads. Reads were aligned in paired-end mode to the hg19 b37 version of the genome using BWA-MEM (Burrows-Wheeler Alignment tool). Local realignment and quality score recalibration were performed using Genome Analysis Toolkit (GATK) according to GATK best practices [11]. Samples were subjected to a series of computational quality control steps to ensure genomic concordance between tumor and normal specimens from control group of normal individuals, detect the presence of tumor DNA in the normal sample, and monitor contamination involving DNA from different patients. Unpaired-sample variant calling was performed on tumor sample and control normals to identify point mutations/single nucleotide variants (SNVs) and small insertions/deletion (indels). MuTect (version 1.1.4) was used for SNV calling and SomaticIndelDetector, a tool in GATKv.2.3.9, was used for detecting indel events. Variants were subsequently annotated using Annovar, and annotations relative to the canonical transcript for each gene (derived from a list of known canonical transcripts obtained from the UCSC genome browser) were reported. Since this tumor was without a matched normal sample, variant calling was performed as: variants with minor allele frequency > 1% in the 1000 Genomes cohort were also removed as they were more likely to be common population polymorphisms than somatic mutations. Annotated SNV and indel calls were subjected to a series of filtering steps to ensure only high-confidence calls were admitted to the final step of manual review. First, prior knowledge from the literature was incorporated in the analysis through a ‘two-tiered’ variant filtering scheme: variants corresponding to known hotspot mutations with extensive supporting evidence in the literature (at least 5 mentions in the COSMIC database) were considered ‘first-tier’ events. These variants were subjected to lower requirements on coverage, number of mutant reads and variant frequency to be considered as high confidence calls. Second, variants detected in more than 20% of a set of historical normal samples (i.e. ≥3 mutant reads and > 1% variant frequency) were considered to be likely artifacts and removed. Third, we employed the following thresholds on coverage depth (DP), number of mutant reads (AD) and variant frequency (VF) for rejecting false positive calls. First-tier variants (i.e. well-characterized hotspot mutations) were considered in a separate class from novel second-tier variants -first-tier variants were filtered using the following criteria: DP ≥ 20X, AD ≥ 8 and VF ≥ 2%, compared to second-tier variants: DP ≥ 20X, AD ≥ 10 and VF ≥ 5%. Variant calls passing these filtering steps and resulting in changes to the protein primary sequence (i.e. non-synonymous: missense and nonsense, splice site, frameshift indel, inframe indel) were subjected to manual review using the Integrated Genomics Viewer (IGV) [12]. This enabled the elimination of additional likely false positive calls (e.g. variants supported by reads with low mapping quality and/or many low-quality bases) produced by sequencing-induced artifacts. Finally, normal variants were excluded by searching the gnomAD.org, a databases annotating human SNPs.

### Mutation validation by sanger sequencing

The E472D missense mutation of PDGFRB was confirmed by genomic locus PCR and Sanger sequencing. Primers used for PCR and Sanger sequencing are: E472D_1F:TCTGCCTCTACCCCATACGC; E472D_1R: GGGCACATTACCAATTAGGCAG.

### *In Situ* detection of phosphorylated PDGFRB in cardiac sarcoma tissue section using duolink fluorescence staining

In situ phosphorylation of PDGFRB was detected by using the Duolink II Red fluorescence staining kit according to the manufacturer's instruction (Olink Bioscience). Formalin-fixed paraffin-embedded tissue sections of cardiac sarcoma and normal heart tissues were deparaffinized, antigen-retrieved by boiling in 2.1% (w/v) citric acid, pH 6.0 buffer, permeabilized with 0.2% (v/v) Triton X-100 in PBS, blocked with kit provided Blocking Solution, and probed with one primary rabbit monoclonal antibody (Cell Signaling) to PDGFRB and another mouse monoclonal antibody to phosphotyrosine (Millipore Sigma), as well as isotype controls (Santa Cruz Biotechnology) (as negative control) at 4°C overnight. The Duolink kit provided PLA probes were added and signal was amplified, labeled by Alexa Fluor 598 (Red). The cell nuclei were counterstained by DAPI (Sigma). Image analysis was conducted using a Zeiss fluorescence microscope.

## DISCUSSION

Primary malignant cardiac tumors are rare, with an incidence of 0.001% ∼0.003%, comprising mainly of sarcomas [13]. The overall prognosis of cardiac sarcomas (CS) is dismal, with surgical resection being the only effective treatment option, as CS does not respond to conventional radiation and chemotherapy. Pathogenic driver gene mutations are largely unknown for this kind of malignancy. Besides angiosarcoma, leiomyosarcoma, rhabdomyosarcoma, undifferentiated sarcoma and myxofibrosarcoma, intimal sarcoma (IS) has recently been identified as the most common type of sarcomas in the heart by the largest cohort of cardiac sarcomas studied to date (100 cases)[2]. According to this study, IS are probably under-diagnosed due to their nonspecific clinical presentation and often mimicking histological appearance to other sarcomas. Efforts have been made to identify specific diagnostic biomarkers for IS, however, because of the rarity of cardiac IS, published studies and data are limited. Five small study series of IS have demonstrated that amplification/activation of PDGFRA/B, EGFR, and MDM2 are common findings in IS [2–5, 14, 15]. Here we report one case of rare cardiac IS in a 70-year old female patient. Using MSK-IMPACT, a next-generation sequencing based genetic profiling assay, we analyzed the mutation status of a panel of 410 key cancer-associated genes, aiming to find a novel diagnostic biomarker and therapeutic molecular target for this neoplasm.

As expected, this case of IS was found in the left atrium and involving mitral valve, where most IS are located (83%) [2]. Pathology examination found histology of a poorly differentiated sarcoma, which consisted of abundant atypical spindle and pleomorphic cells, with high mitotic index. Routine IHC analysis of this tumor showed positive staining of endothelial markers CD31, CD34, smooth muscle marker Calponin, and mesenchymal cell marker vimentin. All of these histological features greatly overlap with those typically seen in UIS [2, 3]. However, the non-specific clinical presentation and high tumor heterogeneity (with a wide spectrum of morphologies), make it very difficult to diagnose IS in practice. IS respond poorly to radiation and chemotherapy, and have no clearly defined molecular diagnostic marker and therapeutic target. Surgical treatments often end up being palliative, and tumor recurrence is unavoidable. The prognosis of most IS remains only 12 months. All of these debacles are due to lack of data and studies, mainly resulting from the rarity of this malignancy. In our current case study, we are the first to use a targeted deep sequencing based comprehensive cancer genes profiling assay to screen 410 of the most important cancer-related genes in this rare tumor. Consistent with previous study results, our gene copy number analysis in this IS case revealed genomic amplification of *PDGFRA*, *MDM2*, *KIT*, and deletion of *CDKN2A* and *CDKN2B,* reiterating such variations as molecular characteristics of IS [2–5, 14, 15].

The nonsynonymous mutations in *PDGFRB*, *KMT2D* and *MPL* are a unique finding in this study. PDGFRA and PDGFRB are class III receptor tyrosine kinases (RTK) functioning in the development of normal connective tissue cells. Aberrant activation of PDGFR signaling due to gene amplification, translocation and activating mutation, has been found in various solid tumors[16]. Limited number of small series studies (7-21 cases), as well as one recent large cohort (100 cases, the largest so far), have identified dysregulated PDGFR (A and B) activity as a possible unique molecular feature of IS [2–4]. However, the underlying genomic alteration causing aberrant expression of PDGFRB remains elusive [2–4]. A recent IS case report by Ito et al uncovered a *PDGFRB* R709H point mutation, which is located in the kinase domain of the protein, implying it may activate PDGFRB signaling [5]. Our current case of IS has identified a novel missense mutation in *PDGFRB*, E472D. We further showed that in this IS tumor, PDGFRB protein was consistently activated (phosphorylated), strongly suggesting E472D be an activating mutation in maintaining aberrant PDGFRB signaling in IS. E472 is located in the immunoglobulin-like (Ig) Domain 5 (D5) of the extracellular part of the receptor, presumably responsible for conveying ligand binding signal down to the intracellular kinase domain, creating protein activation [16]. PDGFR A and B often form heterodimers to activate downstream signaling. The precise pairing of the D4-D5 region of PDGFRB is directly coupled to the activity of intracellular effector tyrosine kinase domain. We speculate that the replacement of glutamic acid with aspartic acid at position 472 might provide a shorter salt bridge for domain-domain interaction, resulting in persistent activation of downstream signaling [16]. Although PDGFRA was found by others to be predominantly amplified/activated in IS, our discovery of a novel, potentially activating PDGFRB mutation, concurrently with PDGFRA amplification, substantiates the forming concept of considering PDGFR signaling as an IS-specific diagnostic biomarker. More interestingly, this insight offers a rationale to use PDGFR small molecule inhibitors, such as imitanib, sunitinib and nilotinib, alone or in combination with other therapies, to treat IS. Of note, although this patient was still alive when this study was conducted, unfortunately she refused to be medicated with any PDGFR inhibitor.

*MPL* gene encodes thrombopoietin receptor, which plays an important role in the development of platelets and megakaryocytes. Mutations of MPL have been identified in myeloproliferative neoplasms [17]. The P453L missense mutation, a novel alteration identified in this case of IS, is located in the Ig FN3 domain of MPL protein, implying the interaction of MPL with other factors might be affected. KMT2D (MLL2), a lysine methyltransferase and global chromatin modifier, is involved in epigenetic gene regulation [18]. KMT2D inactivating mutations have been found in a variety of solid tumors, including cardiac angiosarcoma [19]. Novel A3618P mutation detected in this case of IS does not fall in any functional domain of KMT2D, and its functional consequence remains to be clarified.

A lack of complete functional validation of these genetic alterations is a limitation of our study. Future work will focus on testing the oncogenic potential of these mutations in context of cardiac cells, hoping to identify the driver genetic alteration for IS. In summary, our finding in this case provides novel molecular insights for understanding IS tumorigenic mechanism and, more importantly, for establishing IS standard therapeutic strategy targeting the PDGFR signaling pathway.

### Ethics statement

Investigation has been conducted in accordance with the ethical standards and according to the Declaration of Helsinki and according to national and international guidelines and has been approved by the authors’ institutional review board.

## SUPPLEMENTARY FIGURES


