# Supplementary material for: Activating mutation of PDGFRB gene in a rare cardiac undifferentiated intimal sarcoma of the left atrium: a case report
Source: Oncotarget. 2017 Sep 7;8(46):81709–16. doi: 10.18632/oncotarget.20700 (PMC5655321; doi:10.18632/oncotarget.20700)
Supplement: Supplementary file 1 [file oncotarget-08-81709-s001.pdf]

Activating mutation of *PDGFRB* gene in a rare cardiac undifferentiated intimal sarcoma of the left atrium: a case report

SUPPLEMENTARY MATERIALS

METHOD

Copy number aberrations were identified by comparing sequence coverage of targeted regions in a tumor sample relative to a standard diploid normal sample. Specifically, coverage of targeted regions (exonic and fingerprint regions) was computed using the GATK DepthOfCoverage tool and subsequently adjusted for GC content using a Loess normalization procedure. Normalized coverage values from tumor samples were divid-ed by corresponding values in normal samples, and log-transformed to yield log-ratios. A single tumor sample was compared against multiple normals from the set of control normals to obtain different sets of log-ratio values. The sum-squared log-ratio was com-puted for each normal sample compared against, and was used as a measure of signal-to-noise to select the best comparator normal for the tumor sample analyzed. Log-ratio coverage values were subsequently segmented by circular binary segmentation (CBS), and target regions belonging to the segment cluster with mean segmented log-ratio clos-est to 0 were used to parameterize a null distribution for estimating significance of whole gene copy number events. The following criteria was used to determine signifi-cance of whole gene gain or loss events: fold change > 2.0 (gain) or < -2.0 (loss), $p < 0.05$ (FDR corrected for multiple testing). Matched normal samples were subjected to the same copy number variant calling algorithm. The resulting germline calls were sub-tracted from the total set of copy number calls made on the tumor sample, to ensure the final set of copy number variants from the tumor sample were confirmed to be somatic.

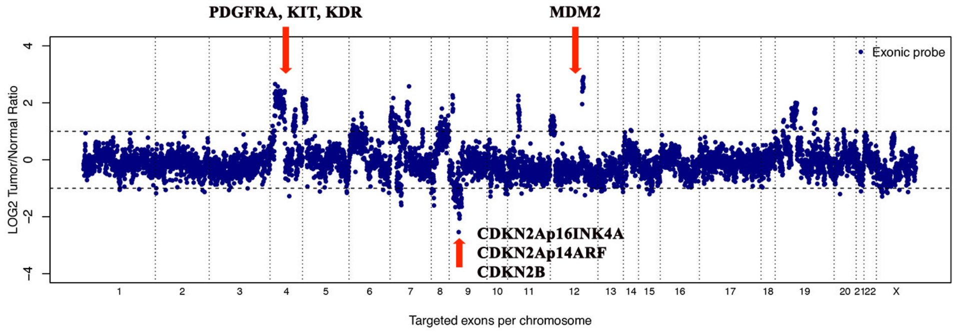

chr	genes	NExons	fc	p.adj
12q15	MDM2	11	6.11*	1.2e−09
4q12	PDGFRA	22	4.07*	4.1e−06
4q12	KIT	21	4.07*	4.1e−06
4q12	KDR	30	4.07*	4.1e−06
5p15	SDHA	15	3.43*	7.9e−05
5p15	TERT	16	3.43*	7.9e−05
19p13	DNMT1	40	3.12*	3.0e−04
19p13	KEAP1	5	3.12*	3.0e−04
7q21	HGF	18	3.11*	3.0e−04
11q13	RPS6KB2	15	3.01*	4.7e−04
19p13	MEF2B	8	2.90*	7.9e−04
7p22	CARD11	24	2.76*	1.3e−03
9p24	CD274	6	2.51*	5.1e−03
4q31	INPP4B	23	2.45*	6.6e−03
12p13	KDM5A	28	2.28*	1.6e−02
12p13	RAD52	11	2.28*	1.6e−02
7p22	RAC1	7	2.23*	2.0e−02
7p22	PMS2	15	1.87	1.6e−01
8q11	SOX17	2	1.78	2.0e−01
8q21	TCEB1	3	1.78	2.0e−01

chr	genes	NExons	fc	p.adj
9p21	CDKN2B	2	−3.47*	8.3e−04
9p21	CDKN2Ap16INK4A	3	−3.47*	8.3e−04
9p21	CDKN2Ap14ARF	2	−3.47*	8.3e−04
9p24	PTPRD	35	−2.10	2.6e−01
7p12	IKZF1	7	−2.09	2.7e−01
9p13	PAX5	10	−2.03	2.9e−01
15q25	NTRK3	18	−1.73	6.7e−01
8p21	NKX3−1	2	−1.68	7.6e−01
7p21	ETV1	11	−1.60	9.9e−01
5q35	FLT4	30	−1.53	9.9e−01
Xp22	CRLF2	6	−1.52	9.9e−01
Xp22	ZRSR2	11	−1.52	9.9e−01
Xp22	EIF1AX	7	−1.52	9.9e−01
Xp11	BCOR	14	−1.52	9.9e−01
Xp11	KDM6A	29	−1.52	9.9e−01
Xp11	RBM10	24	−1.52	9.9e−01
Xp11	ARAF	15	−1.52	9.9e−01
Xp11	GATA1	5	−1.52	9.9e−01
6q26	PARK2	12	−1.50	9.9e−01
18p11	YES1	11	−1.48	9.9e−01

Supplementary Figure 1: Copy number landscape of this case of cardiac IS. The X-axis represents the different chromosomes and the Y-axis quantifies gene copy number tumor/normal log2 ratios.

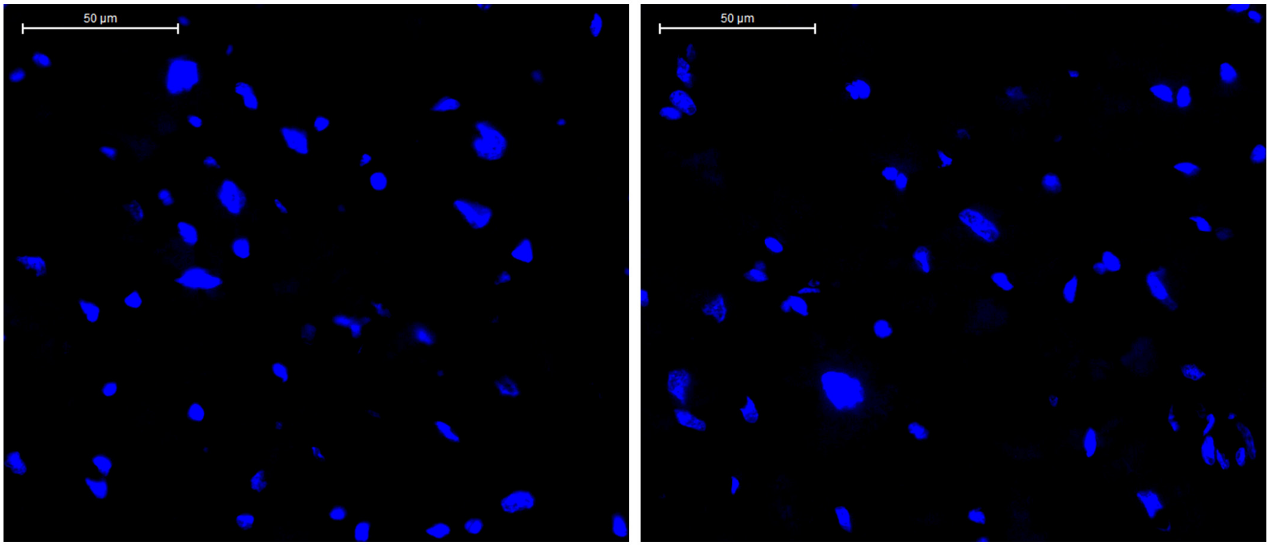

Supplementary Figure 2: Negative control of Duolink staining in normal human heart tissue using mouse and rabbit isotype IgG. (x400). Two representative images of control normal human heart tissue section. DAPI: blue.
